# Evaluation of the residual efficacy and physical durability of five long-lasting insecticidal nets (LLINs) in Senegal

**DOI:** 10.1186/s12936-022-04230-6

**Published:** 2022-07-02

**Authors:** El Hadji Diouf, Mbaye Diouf, Constentin Dieme, Isabel Swamidoss, El Hadji Malick Ngom, Massila Wagué Senghor, Modou Mbaye, Abdoulaye Konaté, Youssouph Coulibaly, Dome Tine, Ibrahima Dia, Ellen Marie Dotson, Ousmane Faye, Lassana Konaté

**Affiliations:** 1 Laboratoire d’Ecologie Vectorielle et Parasitaire, Département de Biologie Animal, Faculté Des Sciences et Techniques, Université Cheikh Anta Diop, Dakar, Sénégal; 2 Institut Pasteur de Dakar, Dakar, Sénégal; 3 Laboratoire de Télédétection Appliquée, LTA/IST/FST/UCAD, Dakar, Sénégal; 4 Division of Parasitic Diseases and Malaria, Centers for Disease Control (CDC) and Prevention, Atlanta, GA USA

**Keywords:** LLINs, Durability, Residual biological efficacy, Retention, Senegal

## Abstract

**Background:**

The preventive and curative strategies of malaria are based on promoting the use of long-lasting insecticidal nets (LLINs) and treating confirmed cases with artemisinin-based combination therapy. These strategies have led to a sharp decline in the burden of malaria, which remains a significant public health problem in sub-Saharan countries. The objective of this study was to determine and compare the residual efficacy of LLINs recommended by the World Health Organization.

**Methods:**

The study was conducted in six villages in two sites in Senegal located in the Sahelo-Sudanian area of the Thiès region, 70 km from Dakar and in Mbagame, a semi-urban zone in the Senegal River Valley. A census was conducted of all sleeping places in each household to be covered by LLINs. Five brands of LLIN were distributed, and every six months, retention rates, net use, maintenance, physical integrity, insecticide chemical content, and biological efficacy were examined for each type of LLIN.

**Results:**

A total of 3012 LLINs were distributed in 1249 households in both sites, with an average coverage rate of 94% (95% CI 92.68–95.3). After 36 months, the average retention rate was 12.5% and this rate was respectively 20.5%, 15.1%, 10%, 7%, and 3% for Olyset Net^®^, Dawa Plus^®^ 2.0, PermaNet^®^ 2.0, NetProtect^®^ and Life Net^®^, respectively. The proportion of LLINs with holes and the average number of holes per mosquito net increased significantly during each follow-up, with a large predominance of size 1 (small) holes for all types of LLINs distributed. During the three-year follow-up, bioassay mortality rates of a susceptible strain of insectary reared *Anopheles coluzzii* decreased in the following net types: in Dawa Plus^®^ 2.0 (100% to 51.7%), PermaNet^®^ 2.0 (96.6% to 83%), and Olyset Net^®^ (96.6% to 33.3%). Mortality rates remained at 100% in Life Net^®^ over the same time period. After 36 months, the average insecticide content per brand of LLIN decreased by 40.9% for Dawa Plus^®^ 2.0, 31% for PermaNet^®^ 2.0, 39.6% for NetProtect^®^ and 51.9% for Olyset Net^®^ and 40.1% for Life Net.

**Conclusions:**

Although some net types retained sufficient insecticidal activity, based on all durability parameters measured, none of the net types survived longer than 2 years.

## Background

Mosquito nets and insecticide-treated materials are the major vector control tools in countries where malaria is endemic [1]. In addition to their effectiveness for vector control [2], these tools, particularly long-lasting insecticidal nets (LLINs), have contributed to reduced malaria morbidity and mortality among children under five years of age [3] who are most vulnerable to malaria infection. These nets have also significantly helped reduce the number of global malaria cases, estimated at 241 million cases and 627,000 deaths in 2020 [4] and the reduction in incidence from 80 to 57 in worldwide and from 363 to 225 in Africa between 2000 and 2019 [5].

In Senegal, the burden of malaria has been significantly reduced over the past decade due to the strengthening of vector control tools (indoor residual spraying and LLINs). The number of malaria cases decreased from 492,253 in 2016 to 354,708 in 2019, a decrease of 28%, and the number of deaths decreased from 526 to 260. The prevalence of *Plasmodium falciparum* infections in children under five years of age has decreased from 1.2% in 2015 to 0.4% in 2019 [6]. However, there is a gradient of transmission intensity, with a gradient in the prevalence from 7.5% in the southeast of the country to almost zero prevalence in parts of the north [7, 8]. Following this success, the National Malaria Control Programme (NMCP) has changed its strategy from control to elimination.

Before the introduction of LLINs, conventional mosquito nets required re-impregnation every 6 to 12 months (the effective period of formulations) or after washing. This was a major constraint in the use of conventionally treated (non-durable) materials in most areas of sub-Saharan African countries, where the re-impregnation rate remained low [9].

In 2002, the Global Malaria to Roll Back Malaria has recommended the use of industrially-impregnated mosquito nets with a long duration bio-efficacy [9] and unaffected by up 20 washes [10–12] over the use of conventional mosquito nets that are pre-treated [5]. Thus, Senegal, in its drive to reduce the number of malaria cases, initiated through the Ministry of Health, Prevention and Social Action, including the NMCP, a vast campaign to assess household access to LLINs. This campaign was followed in 2010 by a mass distribution campaign of LLINs, during which 2,180,000 nets were distributed nationwide to children under five years, giving a coverage rate of 0.31 per household and 0.55 mosquito nets per sleeping space [13].

Although the World Health Organization (WHO) had recommended the use of industrially impregnated LLINs, the assessment of residual insecticide biological activity was often performed over only a short period of time (less than 3 years), not covering the life expectancy specified by the manufacturers (3–4 years), and on a small scale or under laboratory conditions, in experimental huts and semi-field tests [14–16]. Although these types of studies were essential and provided valuable information, they had limitations as to their representation of the effective (real) life of LLINs under field conditions, where they are subject to the effects of local climate, the local handling practice of households, and many other local factors, such as cultural practices (e.g., use for field work, a bed frame corner, tree branch) [17].

Data on LLIN durability under field conditions is needed to determine their turnover rate and actual impact, as well as to provide data to help the manufacturers improve the quality of their nets [17]. Several recent studies of LLINs have addressed their longevity in the field [12, 17–21].

Recently, a proliferation of several brands of LLINs in rural and urban markets has been observed. These come largely from donations from public, private and civil organizations. Despite being beneficial for the protection of populations exposed to vector-borne diseases, a lower quality mosquito nets could inadvertently contribute to the development and spread of vector resistance to insecticides.

The objective of this longitudinal study was to evaluate the durability of five different types of LLINs for their physical integrity and residual insecticide biological activity in households at two sites in Senegal over three years.

## Methods

### Brands of LLIN

A total of five brands of LLINs were distributed to households: NetProtect^®^, PermaNet^®^ 2.0, Dawa Plus^®^ 2.0, Olyset Net^®^ and Life Net^®^ (Table 1). NetProtect^®^ mosquito nets were white, rectangular (length = 190, width = 180 and height = 150 cm) made of 118 denier polyethylene mono filaments and 136 holes/in^2^ mesh. PermaNet^®^ 2.0 mosquito nets were white, rectangular (length = 190, width = 180 and height = 200 cm), made with 75 denier polyester filament tulle and 156 holes/inch^2^ mesh. Dawa Plus^®^ 2.0 were white, rectangular (length = 200, width = 160 and height = 180 cm), made of polyester multi-filament tulle, 75 deniers and 156 holes/inch^2^ mesh. Olyset Net^®^ were white, conical (circumference 1250 cm, height = 250 cm), made with a denier polyethylene mono filament tulle greater than 150 cm and 56 holes/inch^2^ mesh. Life Net^®^ nets were the only mosquito nets made from polypropylene, they were white, rectangular (length = 190, width = 180 and height = 150 cm) made with multi-filament tulle with 110 denier and 156 holes/inch^2^ mesh.

**Table 1:** Characteristics of types of LLINs distributed in study areas

Type of LLINs	Manufacturers	Material	Mesh (holes/square inch2)	Denier	Insecticide	Standard Dose (mg/m^2^)
NetProtect^®^	Intelligent Insect Control, France	Polyethylene	136	118	Deltamethrin	68
PermaNet^®^ 2.0	Vestergaard-Frandsen, Suisse	Polyester	156	75	Deltamethrin	55
Dawa Plus^®^ 2.0	Tana Netting, CO,LTD,Thailand	Polyester	156	75	Deltamethrin	80
Olyset Net^®^	Sumitomo Chemical, Japon	Polyethylene	56	> 150	Permethrin	1000
Life Net^®^	Bayer CropSciences, France	Polypropylene	156	110	Deltamethrin	340

Aside from the Olyset Net^®^ mosquito nets which were treated with permethrin, all other nets distributed were impregnated with deltamethrin. Olyset Net^®^ and Life Net^®^ were donated by OMVS (Senegal River Basin Development Organization) and BAYER AG (Germany), respectively, while the three other brands (PermaNet^®^ 2.0, Dawa Plus^®^ 2.0 and NetProtect^®^) were purchased from vendors approved by manufacturers.

### Study areas and LLINs distribution

**Fig. 1:**
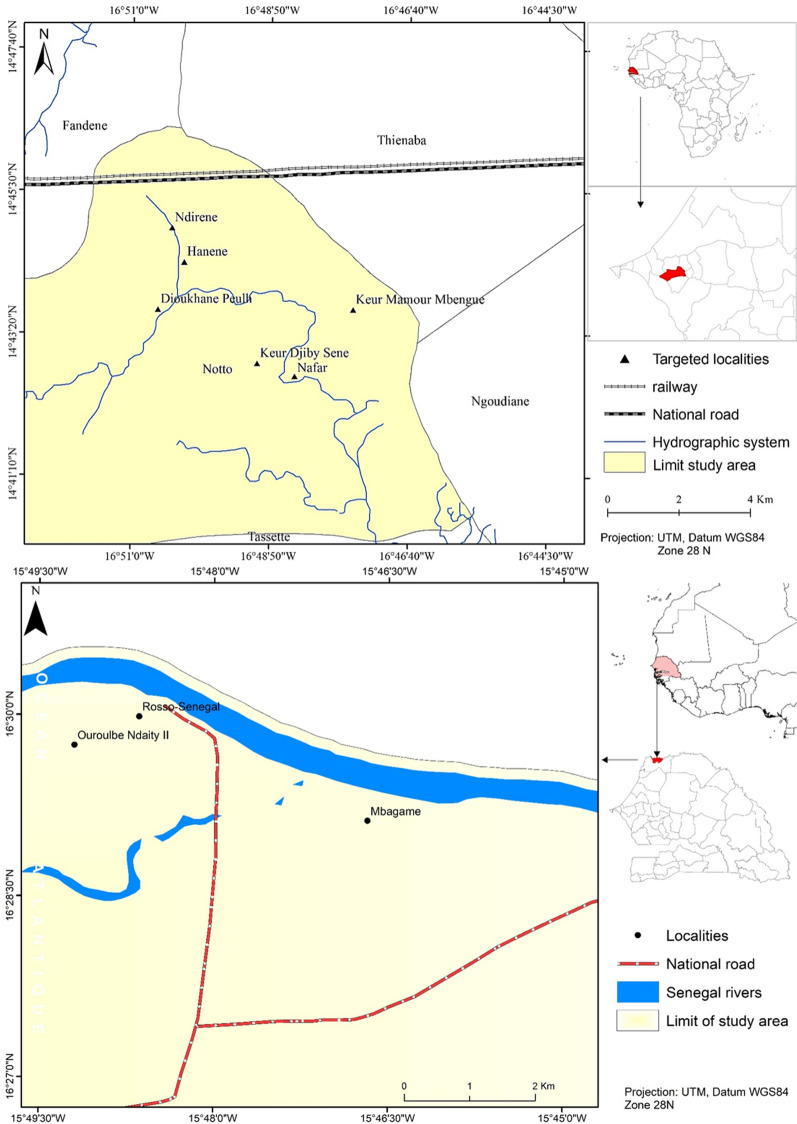
Localities where LLINs were distributed and durability monitored

The study was carried out in 2011 to 2014 in two different bio climatic areas of Senegal (Mbagame: 15° 46′ 59.68’’ W; 16° 29′ 18.23″ N; Thiès: 14° 44′ 23.076″ N; 16° 50′ 6.901″ W): one in the north, in the Senegal River Valley and the other in the west, in the region of Thiès (Fig. 1).

#### Senegal river valley

Olyset Net^®^ mosquito nets were distributed in the village of Mbagame, a semi-urban site located in the Sahelian area in the Richard-Toll health district. The average annual temperatures are around 36–38 °C during the rainy season which lasts two to three months July–September.

This area is characterized by rice crop irrigation, which is a favourable environment for *Anopheles pharoensis*, a species that is present year-round. Its density varies in relation to the water release cycles of the irrigated perimeters. The sporozoite rate in *An. pharoensis* is zero or low [22–24], however, in some locations the biting rate exceeds 100 bites per night (b/p/n). The very high use of mosquito nets throughout the district, particularly in the Walo area (an area at risk of flooding), has been attributed to be in response to this high biting rate [22].

*Anopheles arabiensis* was the most abundant vector in the area, and other species in the *Anopheles gambiae* complex, including *An. gambiae *sensu stricto (*s.s.*), and *Anopheles melas* were also identified but these were detected in very low numbers. Malaria transmission was very low in 2008 with zero prevalence in children less than five years age [11]. In recent years, this has been designated a pre-elimination area by the NMCP, the only one in the country.

#### Thiès

The four other types of LLINs (NetProtect^®^, PermaNet^®^ 2.0, Dawa Plus^®^ 2.0 and Life Net^®^) were distributed in five villages of the Thiès region, in the Sudano-Sahelian area in western Senegal. Four villages are located in the catchment area of the dispensary of Hanène, a village 16.3 km away from the regional capital Thiès, while the fifth is within the catchment of area of the dispensary of Notto Diobass, 6 km from Thiès. All the villages are within the health district of Thiès, located 70 km north of Dakar.

The climate in the Thiès area is Sudano-Sahelian, with a very long dry season from November to July, characterized by low rainfall, low relative humidity (15% on average), warm winds and average annual temperatures ranging from 27 to 32 °C, with a temperature range between 5 and 10 °C. The rainy season was short, lasting three to four months (July/August-October). Average annual rainfall was low and varies widely from year to year (578.3 mm on average) [25]. Thiès is characterized by low malaria endemicity, with an incidence of cases of between 5 and 15 per 1000 inhabitants [26]. Malaria transmission is seasonal and short. It takes place from September to December. The main vector in the area is *An. arabiensis*, whose larval sites are generally created by the rain [27], but shallow wells used for market gardening in certain villages are also very favourable larval habitats.

Mild malaria cases are treated at the household level or at the dispensary in Hanène, on which four of the study villages depend on. The use of insecticide-impregnated mosquito nets remains the main intervention for vector control in the area.

### Identification of sleeping places, estimation of the number of LLINs to be distributed and household survey

Prior to net distribution, a household survey including all residents who had spent the previous night in the household and all sleeping points was conducted in study villages. The gender of the head of the household and any schooling were noted during the survey. Material goods and standard of living indicators (including the presence of electricity, cooking facilities, building structure description) were noted during the survey**.**

Community agents were recruited in each village and after training and orientation, the agents field tested the survey sheet for the identification of sleeping spaces before data collection commenced. For each household, the number of sleeping places to be covered by LLINs prior to distribution was identified and recorded on a survey sheet and then entered in a database. Bedrooms that already had a mosquito net during the survey were not included in the study. Before the distribution of mosquito nets, households participated in an awareness day and coupons containing the number of LLINs to be received were given to future recipients of each village.

### Distribution and use of LLINs

At the distribution in 2011, each LLIN was labeled with a unique identifier and recorded in a database. The labeling was done by concession number, number of households in the concession, as well as the number and rank type(s) of each LLIN introduced. The types of net distributed were randomized to obtain one-third of each type in the households of Hanène and the villages annexed to the health post. Life Net and other types of nets whose durability was not monitored due to disagreement with the local representative, were distributed to households in Ngollar.

At each household visit, information on the use, mode of drying and washing of each net was collected through a questionnaire. Use of net was confirmed in situ by the surveyor. Effective use was defined as use on the night prior to the survey visit.

### Sampling of LLINs for analysis

Every six months during the three-year study period, 28–30 LLINs of each type were randomly selected in the field and replaced with new LLINs, for a total of 900 over the length of the study. The removed LLINs were assessed for physical integrity, residual insecticidal activity and chemical insecticide content analysis as described below.

### Physical integrity of LLINs

The condition of each LLIN was checked every six months. Physical integrity of sampled LLINs was assessed in the laboratory by searching for holes or tears on 170 Dawa Plus^®^ 2.0, 171 NetProtect^®^, 143 Life Net^®^, 178 Olyset Net^®^ and 177 PermaNet^®^ 2.0. Holes were classified as size 1 (0.5–2 cm diameter), size 2 (2–10 cm diameter) or size 3 (diameter > 10 cm) [28]. The proportional hole index (pHI) value was calculated on each LLIN inspected per WHO guidelines [28, 29]. It is the sum of the proportional indexes by categories. The proportional index for a category was the product of the number of holes in category and the index attributed to that size:

$${\text{pHI}} = \# \ {\text{size}}\ 1 \ {\text{holes}}\, \times \, 1 + (\# {\text{size}} \ 2 \ {\text{holes}}\, \times \, 23) + {\text{size}} \ 3 \ {\text{holes}} \, \times \, 196) + (\# {\text{size}} \ 4 \ {\text{holes}} \, \times \,576).$$

The pHI allowed for the classification of nets into three categories according to established thresholds:

Good: 0 ≤ pHI ≤ 64; Damaged: 65 ≤ pHI ≤ 642; Torn: pHI > 642 [13]. Size 4 holes were not considered in this study.

### Residual insecticide biological efficacy

Each semester, bioassays were performed on each of the sampled mosquito nets to determine the residual insecticide effectiveness. Cone bioassays [28] were performed with an Cameroonian strain of *Anopheles coluzzii,* introduced into Senegal used as mosquitoes vector for transmission blocking immunity assay [29]. She is susceptible to DDT, deltamethrin and permethrin, the insecticides present in the different types of distributed LLINs.

For each mosquito net, the bioassays were carried out on five pieces of 30 cm x 30 cm tulle (taken from the four sides and on the roof) and kept in aluminum foil at 4 °C at least one month until testing.

Each section of the mosquito net was inserted between two superimposed rectangular transparent locally produced Plexiglas plates (45 cm × 25 cm). The upper plate used to hold the WHO cones [28] in place had four holes with a diameter equal to the internal diameter of the cones.

With a mouth aspirator, five female mosquitoes aged 3 to 5 days were introduced into the first cone, which was then immediately plugged with a cotton ball. The mosquito exposure time was three minutes per cone. To maintain exact exposure time, mosquitoes were introduced in the second, third, and forth cones at one-minute intervals after the first cone.

An independent timer measured the duration of exposure of female mosquitoes per cone. The experiment was carried out on all five sections of mosquito net, with two cones per piece (5 females tested/per cone) and a total of 50 females tested per mosquito net.

Fifty mosquitoes from the same *An. coluzzii* strain exposed to an untreated mosquito net served as a negative control each test day. For positive controls, three new unused mosquito nets of each type of LLIN were tested using the same method: fifty females per mosquito net, at a rate of 50 females per net. The cones used on one type of mosquito net were also used on the positive controls. At the end of the exposure, the mosquitoes were transferred into a paper cup covered with non-insecticide treated mosquito net, fed with 10% sucrose, and maintained in coolers at 28 °C ± 2 and 80% ± 10% relative humidity. Mosquitoes were then observed at 60 min to determine the knock down (KD 60) and then after 24 h to assess mortality.

### Chemical insecticide content analysis

The residual insecticide content analysis of the removed LLINs at various time points specified in the study was carried out using either high performance liquid chromatography (HPLC) or gas chromatography at the US Centers for Disease Prevention (CDC) in Atlanta, Georgia, USA. Specimens were cut from each bed net using the sampling pattern recommended by WHOPES [28]. For all the net samples, insecticide from five swatches of all the four sides and one from the top were extracted and analysed. Each specimen was trimmed to a 10 cm × 10 cm square (0.010m^2^) using a die cutting in a pneumatic press. During the cutting operation, each specimen was sandwiched between layers of aluminum foil to prevent cross-contamination. The specimen set from each net was analysed as a group to yield an average value of insecticide concentration for the whole net. Chemical analysis was based on methods published by the Collaborative International Pesticides Analytical Council (CIPAC). Deltamethrin analysis of PermaNet^®^2.0 and Dawa Plus^®^ samples was based on CIPAC Method 333 [32–34].

### Extraction of deltamethrin

#### PermaNet^®^ 2.0 and Dawa Plus^®^ 2.0

Each specimen set was weighed and placed in a 125 ml screw capped Erlenmeyer flask and 50 ml of the extraction solvent mixture was added making sure that the net was completely submerged in the mixture. The flask was tightly capped and sealed with paraffin film before placing in an ultrasonic bath for 15 min. Later the flask was shaken in a bath at 25 °C for 30 min at a frequency of 155 cycles per min. The extract was transferred into a chromatographic vial after filtering through a glass syringe fitted with a 0.45 μm reconstituted cellulose syringe filter.

#### Life Net^®^ and NetProtect ^®^

Each specimen set was weighed and placed in a 250 ml round-bottom boiling flask followed by the addition of 95 ml of xylene and 5 ml of a known concentration of internal standard dipropyl phthalate, (2 mg/ml). The flask was fitted with a reflux condenser and heated to boiling for 30 min. After cooling, approximately 2.5 ml of the extract was filtered and transferred to a glass tube and evaporated to dryness under a stream of dry nitrogen for 30 min at 60 °C. A known volume (0.75 ml) of the mobile phase (94/6 (v/v) isooctane/1, 4-dioxane) was used to reconstitute the residue and was transferred to a sample vial using a glass syringe fitted with a 0.45 μm reconstituted cellulose syringe filter. The filtrate was centrifuged (500*g*) for 2 min and transferred into a chromatographic vial after filtering through a glass syringe fitted with a 0.45 μm reconstituted cellulose syringe filter.

#### High performance liquid chromatography (HPLC) analysis

The deltamethrin content was analysed using Agilent 1200 HPLC equipped with a UV detector set at 230 nm and a 150 × 4.6 mm (i.d.) Ascentis Si 5 μm column held at 40 °C. The mobile phase was 94/6 (v/v) isooctane/1, 4-dioxane with a flow rate of 1.5 ml/min. For each extract, three injections of 20 μl were made. Injections, calibrations, and quantification of the deltamethrin content were followed as per the procedure in the Collaborative International Pesticides Analytical Council (CIPAC) 333 [32–34].

#### Extraction of permethrin for Olyset Net^®^

Permethrin analysis of Olyset Net^®^ samples was based on CIPAC Method 331 [31]. Each specimen set was weighed and placed in a 100 ml round-bottom boiling flask, followed by heptane (45 ml) and triphenyl phosphate internal standard (5.0 ml of known concentration in heptane). The flask was fitted with a reflux condenser and heated to boiling for 45 min. After cooling, approximately 1.5 ml of the extract was transferred to a chromatographic sample vial using a glass syringe fitted with a 0.45 μm reconstituted cellulose syringe filter.

#### Gas chromatography analysis

The extracts were analysed using an Agilent 6890 N chromatograph fitted with a 30 m × 0.25 mm (i.d.) fused silica DB-1 capillary column coated with 0.25 μm cross linked polydimethylsiloxane stationary phase. Ultra-high purity nitrogen (1.2 ml/min) was used as the carrier gas. Injector port, column oven, and detector temperatures were 265 °C, 240 °C, and 300 °C, respectively. Flame ionization (FID) was used for analyte detection. Two injections were used for each sample and the results averaged. Permethrin concentration was calculated by comparing permethrin/triphenyl phosphate peak area ratios against a calibration curve generated from solutions containing known permethrin /triphenyl phosphate mass ratios.

### Data entry and statistical analysis

Household survey data were entered on EPI data Entry 3.0, exported to Excel Microsoft office 2010 and analysed with R software. The calculation of proportions and averages was done according to the normal law by 95% Confidence Intervals (CI). Comparisons and proportions were made by Chi-squared tests (*χ*^2^) of homogeneity. Generalized linear models were performed to understand the relationship between mosquito mortality and LLIN washing and mortality bioassay results and insecticide chemical content. The analysis of the condition of the mosquito net, the values obtained for pHI, the torn surface and its shape was used to establish one of the criteria of durability [28]; a mosquito net was in good condition when the pHI was between 0 and 64, is usable when pHI was in 65–642 and was too torn when the pHI was greater than 643 [30]. The acceptable residual efficacy of each type of LLIN was determined based on WHO criteria [30].

Residual efficacy was optimal if the KD 60 or the mortality rate (MR), defined as the number of dead females relative to the total number of mosquitoes exposed per mosquito net was ≥ 95% or ≥ 80%, respectively, after 20 washes in the laboratory or after three years of use [28]. The proportion of mosquito nets meeting optimal bio efficacy levels of KD60 and mortality rates were determined for each type of LLINs by performing cone bioassays in the laboratory. A minimal bio-efficacy criteria of ≥ 75% for KD or ≥ 50% mortality rate was used to determine the efficacy status of LLINs [28]. The MR was corrected by the Abbott formula [35] if the mortality rate for controls was less than 20% and the bioassay was redone if it was greater than 20%. Generalized linear regression models were run to estimate the relationship in mortality rate between washed and non-washed mosquito nets.

The retention rate was calculated for each type of LLIN at each follow up period. It was defined as the ratio of the number of the original study mosquito nets available in households to the total number of mosquito nets initially distributed. The mosquito nets declared lost but found in the following survey were included in the calculation of the retention rate of the previous semester.

### Ethical review

The study was approved by National Committee of Ethics and Health Research in Senegal (CNERS) under number 175 and the Office of the WHO in Senegal.

## Results

A total of 1,249 households were enrolled in both study areas. On average, 92.7% (95% CI 90–94) of heads of households were men, with an average 53 years of age. This average percentage was 78.2% in the Mbagame area with an average age of 52 years (95% CI 49–55) and 94.8% in the villages around Thies with a mean age of 53 years (95% CI 51.6–54.4) (P < 0.001). The average percentage of heads of households with any schooling was 38.4% (95% CI 34–42). The gender breakdown was 96% for men (n = 456) and 4% for women (n = 19).

Of 604 households, 37.7% (228/604) had means of transportation among which 68% (95% CI 61–74) used carts while only 14% (95% CI 8–20) owned cars. The overall average percentage of households with electricity was 31% (95% CI 27–35). In Mbagame village, 87.2% households had electricity compared to an average of 22.6% for the study villages near Thies (*χ*^2^ = 132.4377, df = 1, P < 0.0001). Wood was the main form of fuel for cooking in 97.7% of households in both study areas.

### Mosquito net coverage

A total of 3012 nets were distributed in 1249 households (Table 2). The overall average household coverage, 94% (95% CI 92.68–95.3), was 68.2% (95% CI 63.63–72.77) and 92% (95% CI 90–94) in Mbagame and Thies area villages, respectively. Moreover, the average percentage of households receiving only one mosquito net was 26% (95% CI 23–28) in all areas. The average percentage of households in Mbagame and Thies receiving only one mosquito net was estimated as 41.8% (95% CI 36.9–46.8) and 17.4% (95% CI 15–20.6), respectively.

**Table 2:** Number and type of LLINs distributed per area

Type of LLINs	Number distributed	
	Thiès	Mbagame
DawaPlus^®^ 2.0	609	0
LifeNet^®^	391	0
NetProtect^®^	578	0
OlysetNet^®^	0	853
PermanNet^®^ 2.0	581	0
Total	**2159**	**853**

The global coverage of sleeping spaces (3,012/3,840) was 78% (95% CI 77–78). However, the sleeping space coverage in Mbagame area was estimated at 68.2% (95% CI: 65–70), and 95.3% (95% CI 94–96) in the villages surrounding Thiès.

### Rate of sampling mosquito nets, retention of LLINs

**Fig. 2:**
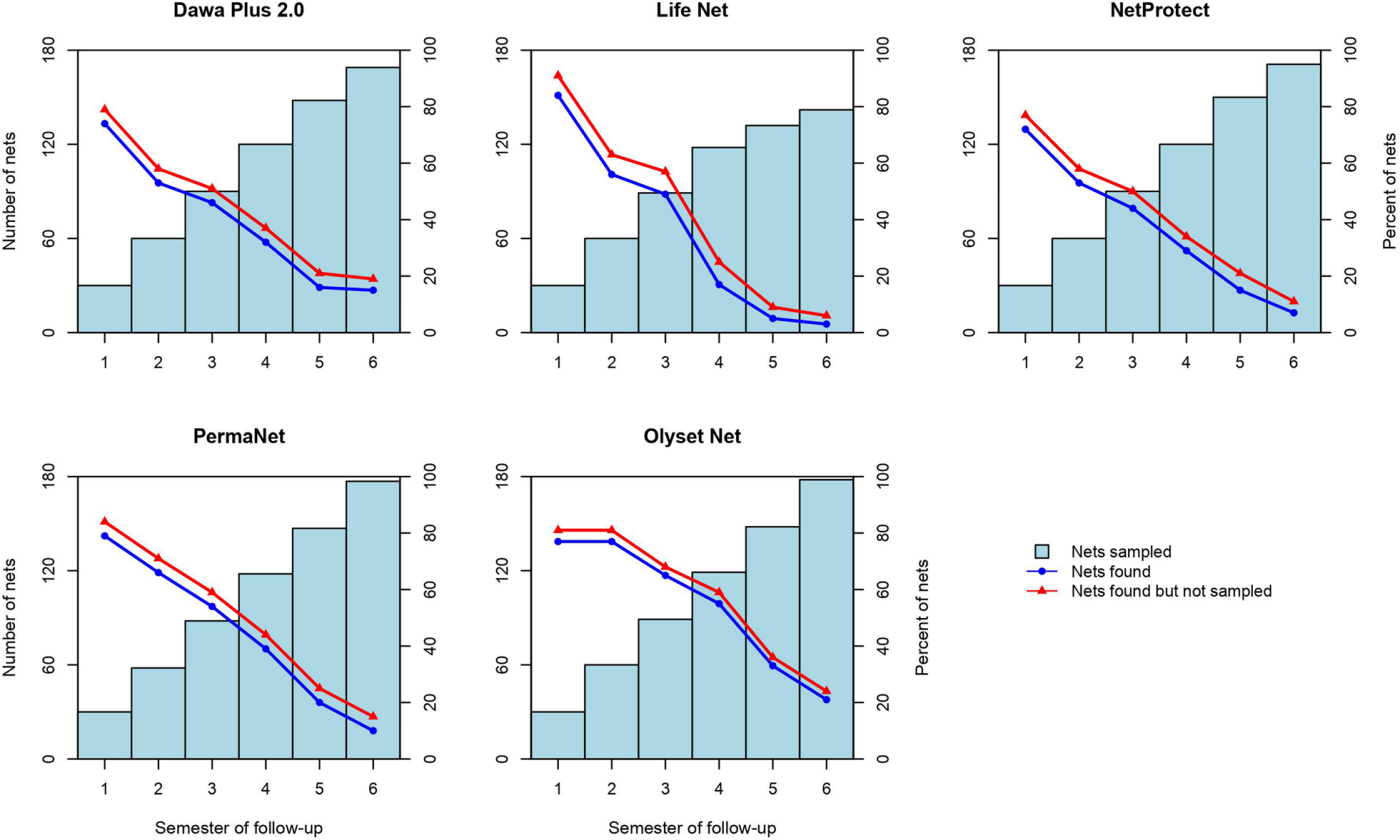
Retained proportions not removed (red), retention after sampling (blue) and cumulative number of nets removed by type and semester after 3 years

From the beginning of the study in 2011 to the end in 2014 (6 semesters), 839 mosquito nets (all types) were removed representing 93.2% of the expected 900 LLINs to be removed (Fig. 2, Table 3). Of the 180 mosquito nets of each brand expected to be sampled, 94.4%, 79.4%, 94.4%, 98.9% and 98.3% of the Dawa Plus^®^ 2.0, Life Net^®^, NetProtect^®^, Olyset Net^®^ and PermaNet^®^ 2.0, respectively, were removed from the field. The number of mosquito nets sampled was less than the 28–30 units expected in the 5th and 6th semester for Life Net^®^, and in the 6^th^ semester for Dawa Plus^®^ 2.0 and NetProtect^®^. At the end of the study, the retention rate of all five LLIN types was 12.5% (95% CI 11.4–13.7) based on the total number of nets distributed and was relatively better for Olyset Net^®^ at 20.5% (95% CI 17.8–23.4) and to a lesser extent for Dawa Plus^®^ 2.0 with 15.1% (95% CI 12.4–18.2). With more than half of the mosquito nets not found after only two semesters of follow-up, the average percentage of Life Net^®^ found in households fell to about one in five mosquito nets distributed remaining in the third semester.

**Table 3:** Physical integrity of the LLINs inspected in the laboratory per type and semester after three years

Semesters	Types	N	General state					
			Good condition		Serviceable condition		Tear	
			N	%	N	%	N	%
1								
	Dawa Plus^®^ 2.0	30	21	70	2	7	7	23
	Life Net^®^	30	28	93.3	1	3.3	1	3.3
	NetProtect^®^	30	26	86.7	0	0	4	13.3
	Olyset Net^®^	30	13	43.3	7	23.3	10	33.4
	PermaNet^®^ 2.0	30	19	63.3	0	0	11	36.7
2								
	Dawa Plus^®^ 2.0	30	18	60	5	16.7	7	23.3
	Life Net^®^	30	24	80	0	0	6	20
	NetProtect^®^	30	22	73.3	2	6.7	6	20
	Olyset Net^®^	30	13	43.3	10	33.4	7	23.3
	PermaNet^®^ 2.0	28	10	35.7	2	7.1	16	57.2
3								
	Dawa Plus^®^ 2.0	30	15	50	5	17	10	33
	Life Net^®^	29	18	62	2	7	9	31
	NetProtect^®^	30	18	60	12	40	0	0
	Olyset Net^®^	28	7	25	9	32	12	43
	PermaNet ^®^2.0	30	19	63	2	7	9	30
4								
	Dawa Plus^®^ 2.0	30	1	3	20	67	9	30
	Life Net^®^	29	25	86	3	10	1	3
	NetProtect^®^	30	14	47	3	10	13	43
	Olyset Net^®^	30	0	0	24	80	6	20
	PermaNet ^®^2.0	30	11	37	11	37	8	27
5								
	Dawa Plus^®^ 2.0	29	10	34	10	34	9	31
	Life Net^®^	15	15	0	0	0	0	0
	NetProtect^®^	30	16	53	3	10	11	37
	Olyset Net^®^	30	5	17	18	60	7	23
	PermaNet^®^ 2.0	29	16	55	6	21	7	24
6								
	Dawa Plus^®^ 2.0	21	1	5	18	86	3	14
	Life Net^®^	10	0	0	0	0	10	100
	NetProtect^®^	21	15	71	1	5	5	24
	Olyset Net^®^	30	2	7	24	80	4	13
	PermaNet ^®^2.0	30	15	50	10	33	5	17

N: number of samples

### Use and maintenance of LLINs

The regular use of the mosquito nets (use the night before the survey) at the end of the follow up was 23.3%, 38.5%, 7.3%, 78.3% and 16.1% for the Dawa Plus^®^ 2.0, Life Net^®^, NetProtect^®^ Olyset Net^®^ and PermaNet^®^ 2.0 types, respectively. For the NetProtect^®^ this rate was statistically lower compared to the Life Net^®^ and Olyset Net^®^ (Fisher's exact test, OR: 0.13; 95% CI: 0.017–0.83, P = 0.014; Fisher’s exact test, OR: 0.022; 95% CI 0.017–0.83, P < 0.001).

Dawa Plus^®^ 2.0 and PermaNet^®^ 2.0 used the night before the survey consistently exceeded 50% during the first four semesters. For Olyset Net^®^, this rate was always above 60% during the three years of follow-up. In contrast, the NetPotect^®^ and Life Net^®^ types were the least used in households. Their regular use rates were only satisfactory in the first two semesters following LLINs distribution, with 52.6% (95% CI 47.7–57.5) for NetProtect^®^ in semester one and 53% (95% CI 46–59.6) for Life Net^®^ in the second.

For all LLIN types distributed, only 19% (95% CI 18–20) were washed. The results obtained with washing habits showed that 66% (95% CI 64–69) of the washed mosquito nets were washed with warm water. In addition, 73% (95% CI 71–76) of the cases used local soap and 50% of the mosquito net was dried in shade after the washing.

### Physical integrity

At least 83% (95% CI 80–90) of mosquito nets had holes (all types of holes) at the end of the study. Except the Life Net^®^, the average number of holes increased with time for all LLINs. For the LLINs inspected in laboratory, at the end of the 6th semester of follow-up, the majority of them had holes, with average proportions of 95.2% 95%, 93.3%, 100% and 60% for Dawa Plus^®^ 2.0, NetProtect^®^ PermaNet^®^ 2.0, Olyset Net^®^ and Life Net^®^, respectively. The average number of holes remained relatively low for Life Net^®^ (Fisher's exact test, OR: 12.02; 95% CI 0.96–684.89, P = 0.027).

**Fig. 3:**
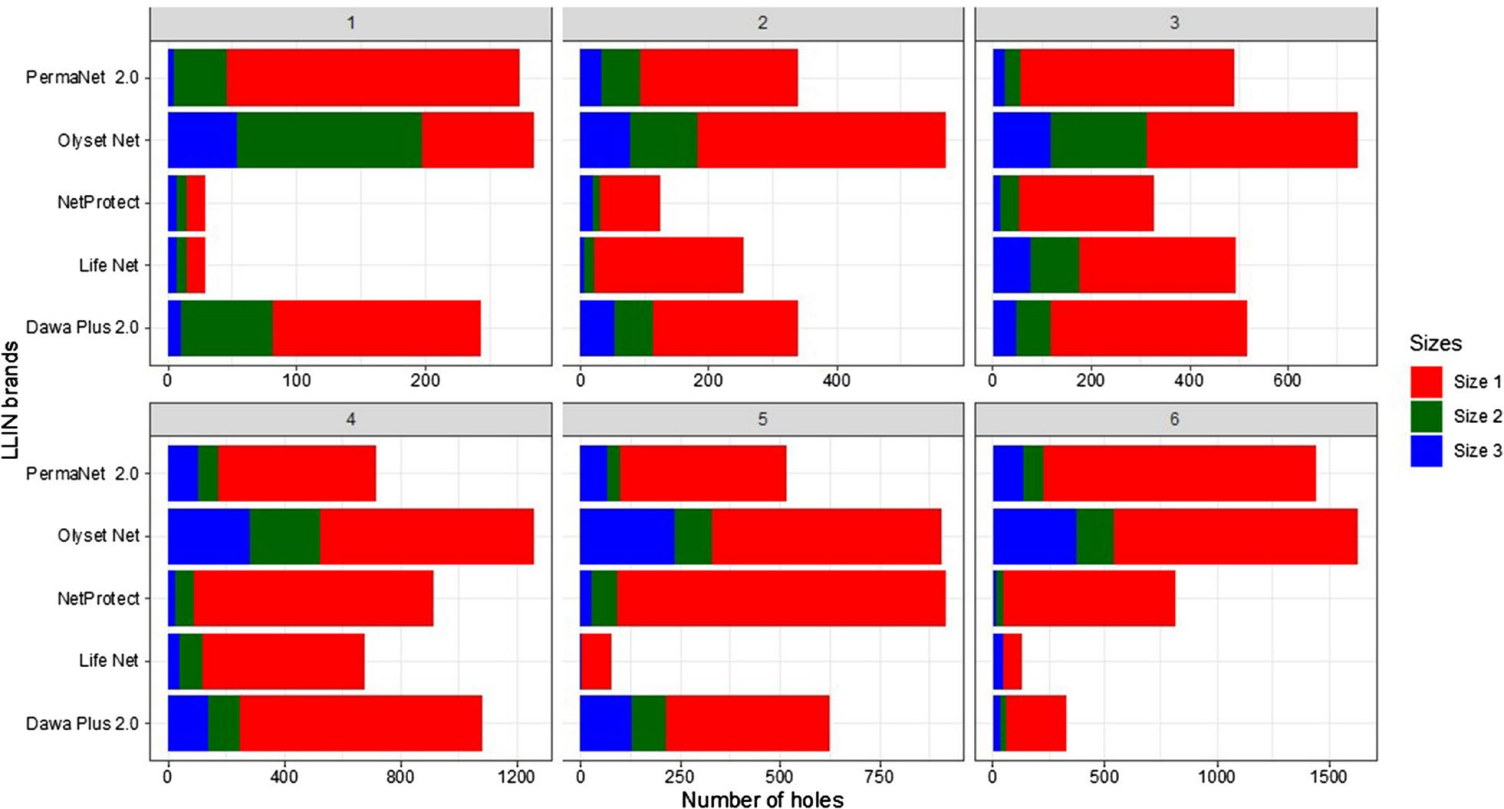
Categories of holes by type and semester after 3 years. Size 1, size 2, size 3

At each semester, size 1 holes (Fig. 3) were predominant for all types of mosquito nets. The average number of holes (all types) per mosquito net was 39 per LLIN at the end of six semesters of study. It was 13, 39, 15, 48 and 54 respectively for Life Net^®^, NetProtect^®^ Dawa Plus^®^ 2.0, PermaNet^®^ 2.0 and Olyset Net^®^, respectively. Only NetProtect^®^ and PermaNet^®^ 2.0 types were still in good condition at the end of the three-year study (Table 3). Proportions of mosquito nets in good condition in households were 71% (NetProtect^®^) and 50% (PermaNet^®^ 2.0) in both mosquito nets.

### Residual insecticide biological efficacy

A total of 839 LLINs were assessed for insecticidal efficacy of the LLINs (Table 4). The KD60 was above 95% for all LLIN types tested in the first four semesters but at the end of the 5th follow-up, three LLIN types (NetProtect^®^ Olyset Net^®^ and PermaNet^®^ 2.0) had a KD60 of less than 95% (Table 4). For all LLIN types taken together, 50.6% (95% CI 47–54) of LLINs had a mortality rate (MR) greater than or equal to 80% at the three-year follow-up. The results varied depending on net type and semester. For NetProtect^®^ and Dawa Plus^®^ 2.0, 56% (95% CI 37–74) and 51.7% (95% CI 32–70) of LLINs had a MR less than 80% in the first five follow-up visits. Conversely, Life Net^®^ and PermaNet^®^ 2.0 types had MR which consistently exceeded 80% throughout the follow-up. For the Olyset Net^®^, the MR was higher than 80% only in the first follow-up at 96.6% (95% CI 82.7–99) and was statistically different between semesters (Fisher's exact test, OR: 53.50735; 95% CI 6.94–2445.45, P ˂ 0.001). Untreated mosquito nets used as negative control had no effect on the mosquitoes with zero mortality and KD60.

**Table 4:** Cone bioassay results using *An. coluzzii* (strain sensitive) per semester and types of LLINs

Net type	Semesters KD60 (n = samples analyzed) [95% CI]					
	1	2	3	4	5	6
Dawa Plus^®^ 2.0	96.4 (n = 30) [82.7–99]	96.6 (n = 30) [82.7–99]	100 (n = 30) [88.4–100	96.6 (n = 30) [82.7–99]	100 (n = 29) [88–100]	95.2 (n = 21) [76.2–99]
NetProtect^®^	100 (n = 30) [88.4–100]	96.6 (n = 30) [82.7–99]	100 (n = 30) [88.4–100]	100 (n = 30) [88.4–100]	93.3 (n = 15) [77.9–99]	95.2 (n = 21) [76–99.8]
Life Net^®^	100 (n = 30) [88.4–100]	100 (n = 30) [88.4–100]	100 (n = 29) [88–100]	100 (n = 29) [88–100]	100 (n = 30) [78–100]	100 (n = 10) [69–100]
Olyset Net ^®^	100 (n = 30) [88.4–100]	96.6 (n = 30) [82.7–99]	100 (n = 28) [88.4–100]	100 (n = 30) [88.4–100]	93.3 (n = 30) [77.9 -99.1]	95.2 (n = 30) [76–99.8]
PermaNet^®^ 2.0	100 (n = 30) [88.4–100]	96.6 (n = 28) [82.7–99]	100 (n = 30) [88.4–100]	100 (n = 30) [88.4–100]	93 (n = 29) [77.9–99,1]	95 (n = 30) [76.1–99.8]
Mortality rate 95% CI						
Dawa Plus^®^ 2.0	100 [88.4–100]	93.3 [78- 99]	96.6 [83–99]	93.3 [78–99]	51.7 [33–71]	100 [84–100]
NetProtect^®^	66.7 [47–82]	93.3 [78–99]	93.3 [78–99]	86.7 [69–96]	56.6 [37–74]	80 [58–94]
Life Net^®^	100 [88–100]	100 [88–100]	100 [88–100]	100 [88–100]	100 [78–100]	100 [69–100]
Olyset Net ^®^	96.6 [83–99]	20 [7_38]	28 [13–49]	13 [4–30]	46 [28–65]	33.3 [17–52]
PermaNet^®^ 2.0	96.6 [83–99]	82 [63–93]	100 [88–100]	100 [88–100]	93 [77–99]	83 [65–94]

N: number of nets collected and analyzed per semester; KD60: average knock down at 60 min; CI: 95% confidence interval

### Chemical insecticide content analysis

**Fig. 4:**
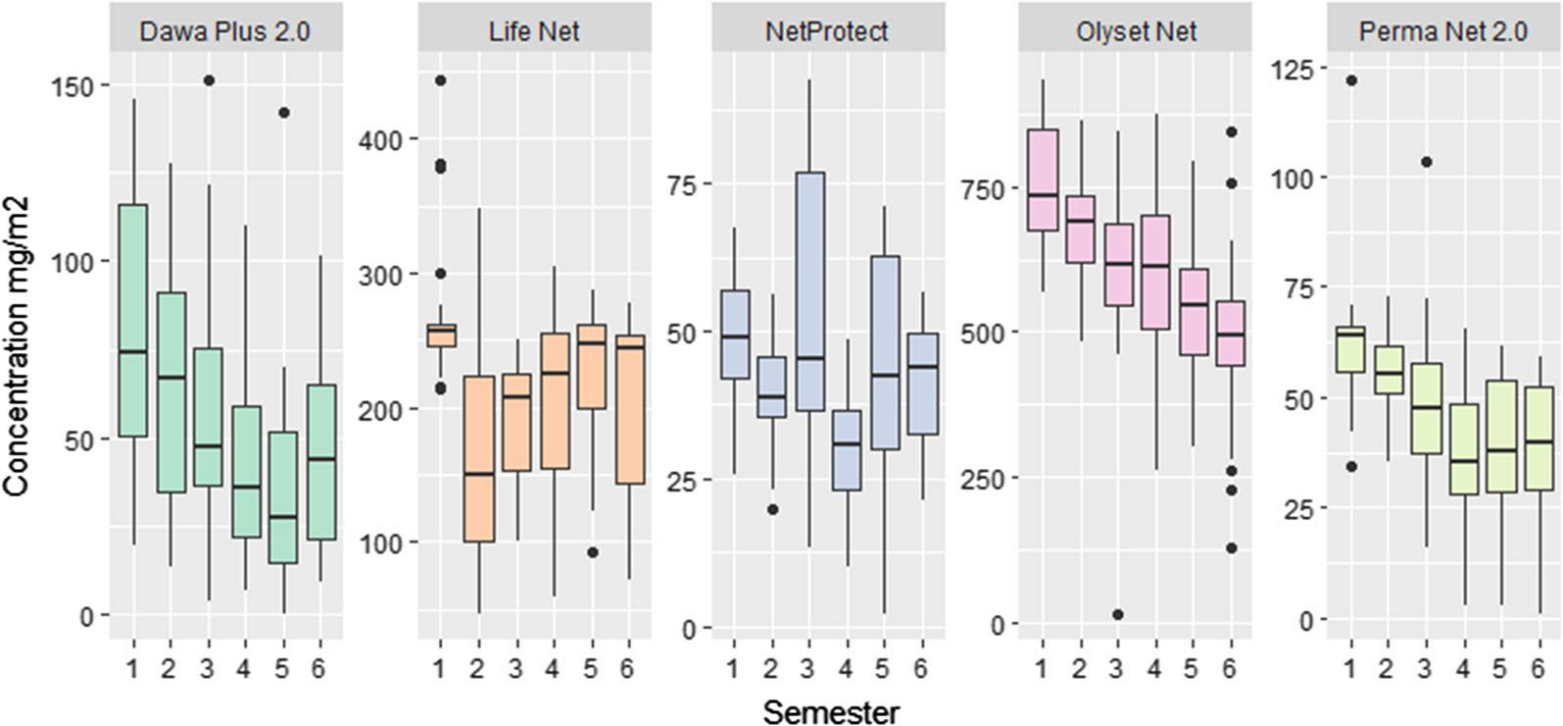
Insecticide concentration in mg/m^2^ according to the type LLINs and semester

The analysis of the average insecticide content for Dawa Plus^®^ 2.0, Olyset Net^®^ and PermaNet^®^ 2.0 showed a gradual reduction after follow-up; unlike Life Net^®^ and NetProtect^®^ where the decrease in insecticide content was generally low and less gradual depending on the semesters (Fig. 4). For the Dawa Plus^®^ 2.0 and PermaNet^®^ 2.0 LLINs, the global median deltamethrin content per LLIN at the end of follow-up was 49.6 mg/m^2^ with IQR 56.9 mg/m^2^ and 49.5 mg/m^2^ with IQR 23.47 mg/m^2^, respectively. At 36 months (end follow-up), it was 74.5 mg/m^2^ (IQR 65.5 mg/m^2^) and 43.7 mg/m^2^ (IQR 43.9 mg/m^2^) for Dawa Plus^®^ 2.0 and 64.2 mg/m^2^ (IQR 10.3 mg/m^2^) and 39.7 for PermaNet^®^ 2.0.

For Olyset Net^®^, the overall median permethrin content was 620.75 mg/m^2^ (IQR of 198.93/m^2^). Specifically, the median permethrin content was 730 mg/m^2^ (IQR 177.8 mg/m^2^) at six months and 488.8 mg/m^2^ (IQR 139.6 mg/m^2^) at 36 months, respectively***.***

#### Mortality rate associated with insecticide content

**Fig. 5:**
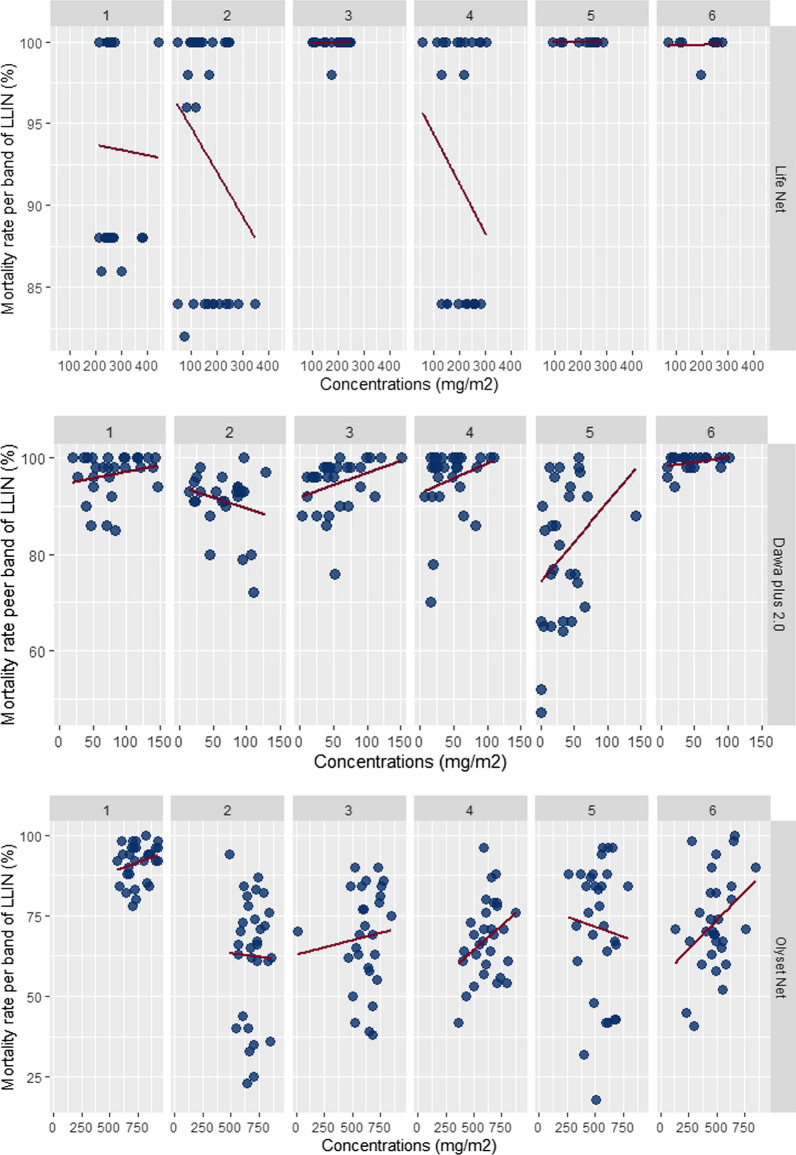

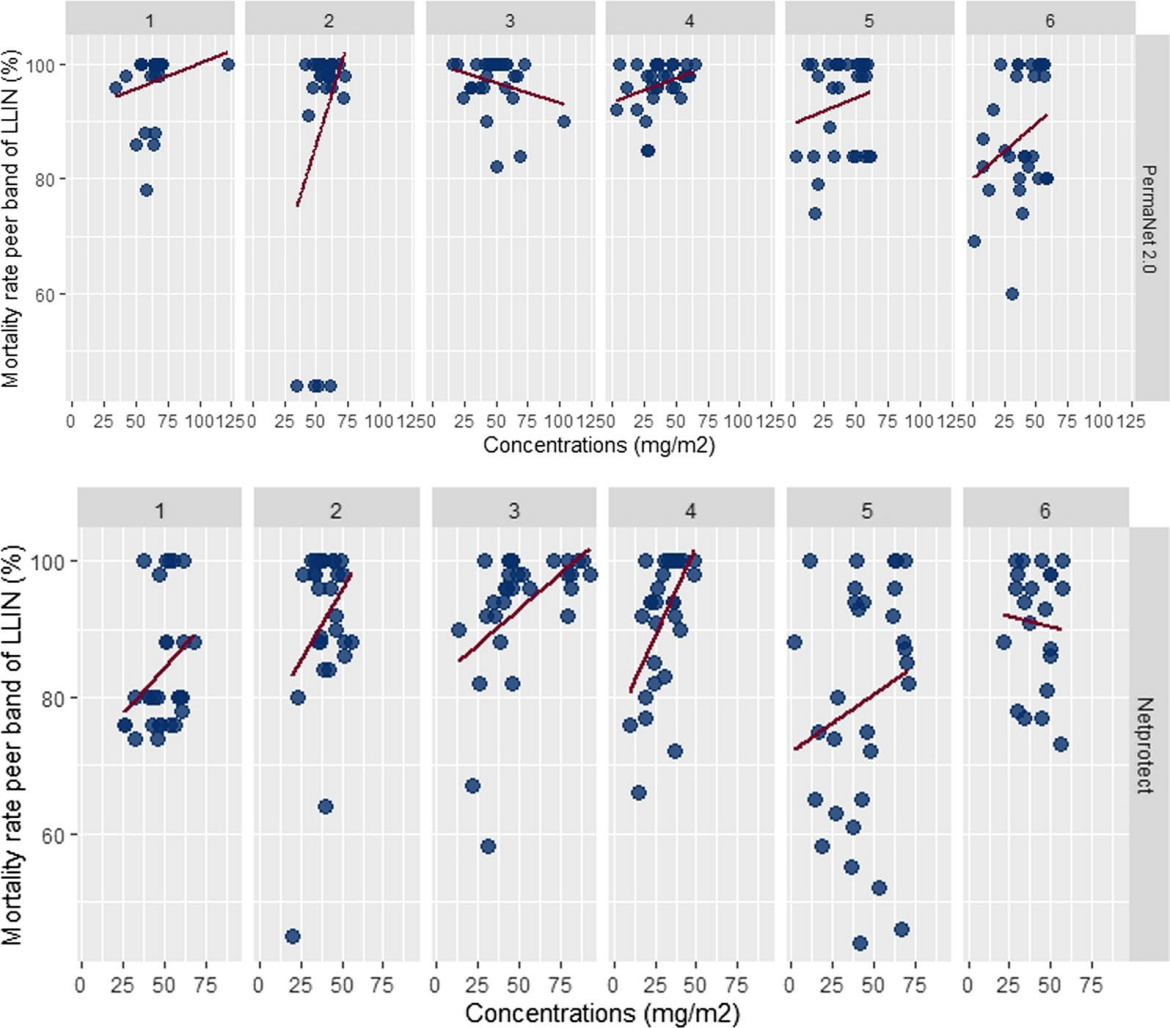
Correlation of chemical and bioassay results by type of LLIN and semester

Residual efficacy greater than 95% knock down was probably correlated with the content of insecticide. The overall correlation coefficients were: r = 0.08, r = 0.08, r = 0.22, r = 0.09 and r = 0.08 for Dawa Plus^®^ 2.0, NetProtect^®^ Life Net^®^, Olyset Net^®^ and PermaNet^®^ 2.0, respectively (Fig. 5). For mortality rate, the correlation coefficient was more important for Life Net^®^(r = 0.43) when compared to other LLIN types.

The generalized linear regression models have indicated that the mortality rate was significantly decreased with washing mosquito nets (Table 5). A significant difference (p < 0.001) in mortality rate was observed between washed and unwashed mosquito nets. This mortality rate was also associated with the average the amount of insecticide content present on the net.

**Table 5:** Multiple regression washing and efficiency of LLINs

Parameter	95% CI for odds Ratio			
	p-value	Lower	Odds ratio	Upper
Mortality (Intercept)	< 2.10^–16^	–	–	–
Ever Washed	2.18 10^–11^	0.89	0.91	0.94
Insecticides	2 10^−16^	1.0005	1.0006	1.0007

CI: Confidence interval

## Discussion

This prospective study, conducted on 3,012 LLINs distributed in two epidemiological areas of Senegal, reveals an overall retention rate of 12.5% at the end of three years. This retention rate is relatively low and could lead to a resurgence of malaria if more nets are not distributed in the area before the next transmission period. In Uganda and Zambia [20, 36], similar results have been reported; however, much higher retention rates were observed in [19, 37, 38] Rwanda, Kenya and Nigeria.

The low retention rate of the LLINs deployed in our study could be explained by the mass distribution campaign implemented in the area during the fifth semester or by the gifting of nets to relatives or friends observed during the first six months. A study has shown that, in Senegal, loss due to moving away is the most important during the first month post-distribution [39]. However other attrition factors related the loss and deterioration of LLINs are observed in the field during surveys. Among the LLIN types, significant differences were observed in the rates of retention. Olyset Net^®^ and Dawa Plus^®^ 2.0 LLINs were the most retained, with 20.5% and 15.1%, respectively. The better retention rate of Olyset Net^®^, the only LLIN type distributed in Mbagame site, may be explained by its very high usage by populations in previous studies (60%), the strong biting behaviour of mosquitoes, especially *An. pharoensis,* in the valley [22] and the culture of net usage.

The results on the physical integrity of LLINs showed that for all types, 83% of LLINs inspected had holes. A similar proportion of holes have been found on the LLINs introduced in Cameroun, Zambia and Tanzania [20, 21, 40]. The holes of size 1 were the most dominant during our follow-up, which is similar to what has been found [21] in Cameroon. In Sub-Saharan countries, studies revealed an average of 30 holes per LLIN; 12 holes in Burundi and 12.5 holes per LLIN in Côte d’Ivoire [41]. Our study recorded an average of 39 holes per LLIN after follow-up. However, for Olyset Net^®^, we found 54 holes per LLIN, this average number of holes is higher than those obtained in Kenya [21]. Furthermore, a study in Ethiopia reported 21 holes per LLIN for Dawa Plus^®^ 2.0 and PermaNet^®^ 2.0 [18].

The relationship between the physical durability of mosquito net and the values of the pHI (Table 3) reflects the longevity (deterioration) of the LLINs. This relationship was measured by the median index and revealed that more than half of Olyset Net^®^ type LLINs were in poor condition compared to Dawa Plus^®^ 2.0; all thirty Olyset Net^®^ sampled at 36 months had lost their physical barrier. This result corroborates those found recently in Senegal [39].

During the site visits to remove the LLIN samples, the households explained to the study team some of the challenges in maintaining nets in good condition such as children’s restless sleeping behaviour, the size of the nets being too small for the beds and conducive to stretching, among other factors.

Indeed, with a lower number of holes per LLIN, a lower pHI and similar washing and drying habits, Dawa Plus^®^ 2.0 and PermaNet^®^ 2.0 LLINs were significantly more resistant to tears than Olyset Net^®^ LLINs as found on an study in Senegal [42]. However, a study in Zambia did not find statistical difference in durability between LLINs PermaNet^®^ 2.0 and Olyset Net^®^ [21].

The NetProtect^®^ nets were the least used of all net types. Households reported to the survey team that the rough material of this LLIN and it smaller size, may explain why they were not used and explain their better preservation of physical integrity. Similarly, users of Life Net type reported frequent itching and these nets also presented good physical integrity at the end of the study. In this study, NetProtect^®^ LLINs were found under beds and households noted that this is to control other pests or disease vectors such as bedbugs, fleas. This fact was likely to increase household retention, but the rate of use the night before follow-up was consistently the lowest throughout the survey.

The insecticidal effectiveness in the study was conducted according to WHOPES recommendations. A decrease in the mortality rate of mosquitoes exposed to Olyset Net^®^ from 96.6% to 20% was observed between the first semester and second semester and remained low to the end of the follow-up compared to other types of LLINs. It was found that lethal effect of Olyset Nets^®^ was less than that of PermaNet^®^ 2.0 [41]. In Olyset Net^®^, the optimal biological efficacy (≥ 95% or ≥ 80%) was observed in the first semester. This could be explained by the low level of permethrin after three years, which was lower than the WHOPES recommended dose of 1000 mg/m^2^. Results reported in the literature have suggested that a content of permethrin greater than 1000 mg/m^2^ and 10 mg/m^2^ deltamethrin typically result in a KD60 rate of 75–95% [20].

However, it would be difficult to explain the findings of bioassay test obtained with Olyset Net^®^ in the first semester with concentrations well below 1000 mg/m^2^. The relationship between the proportion of effective nets (bio-efficacy ≥ 75% for KD or ≥ 50% mortality rate at least) and insecticide content varied considerably between net types. This relationship is reflected in a decrease in net performance in the bioassays. However, despite the report results on bioassay and insecticidal content relationship, it would be important to investigate precisely the concentrations required to accurately define the efficacy criteria (optimal and minimal). Multiple regression results also reported that mosquito net washing reduced the mortality rate compared to unwashed LLINs. The limitations of this study were mainly related to the introduction of universal coverage during the fifth semester while the study was still in progress. This universal coverage appeared to reduce the retention rate of the study nets.

## Conclusions

The evaluation of LLINs after three years of use revealed a loss of integrity of almost all types of nets distributed. Despite this loss of integrity, biological efficacy was maintained for the Dawa Plus^®^ 2.0, PermaNet^®^ 2.0 and Life Net^®^ brands.

## Data Availability

The data used and analysed during the current study are available from the corresponding author on reasonable request.

## Abbreviations

CI 95%: Confidence interval 95%
CIPAC: Collaborative international pesticides analytical council
KD60: Proportion of mosquitoes knocked down at 60 min post-exposure
HPLC: High performance liquid chromatography
IQR: Intra quartile range
LLIN: Long-lasting insecticidal nets
pHI: Proportional hole index
WHO: World Health Organization
WHOPES: World Health Organization pesticide evaluation scheme
UV: Ultraviolet
PVC: Polyvinyl chloride
CDC: Centers for disease control and prevention
MR: Mortality rate
CNERS: National research council for health
OMVS: Senegal river basin development organization
NMCP: National malaria control programme
P: Probability
OR: Odd ratio
